# Study on the Microstructure Evolution of CuxNi2.7Mn Steel During Processing with Different Copper Contents

**DOI:** 10.3390/ma19091906

**Published:** 2026-05-06

**Authors:** Yingchi Zhang, Jing Guo, Chengsheng Yu, Pengyu Wen, Lili Li

**Affiliations:** 1 School of Materials and Metallurgy, University of Science and Technology Liaoning, Anshan 114051, Chinachengsheng_yu9401@163.com (C.Y.); 2 State Key Laboratory of Advanced Metallurgy, University of Science and Technology Beijing, Xue Yuan Lu 30, Beijing 100083, China; wenpy936@ustb.edu.cn

**Keywords:** Cu content, mechanical properties, CuxNi2.7Mn steel, heat treatment

## Abstract

Copper-bearing low-carbon high-strength steels are widely employed in marine engineering. However, the microstructural homogeneity, strength–toughness matching, and low-temperature toughening mechanisms of such steels at high copper contents remain unclear. Existing studies have predominantly focused on the Cu content range of 1–2 wt.%, lacking systematic comparisons regarding microstructural evolution and property regulation throughout the entire rolling-heat treatment process at higher Cu levels. To clarify the influence of Cu content on the microstructural evolution and mechanical properties of CuxNi2.7Mn steels during processing and heat treatment, and to fully exploit the Cu precipitation strengthening effect while suppressing its embrittlement drawback, this study investigates CuxNi2.7Mn steels with Cu contents of 1.35 wt.%, 3.1 wt.%, and 6 wt.%. The specimens were fabricated via vacuum melting and two-stage rolling. Combining in situ observation using a high-temperature laser confocal microscope, optical microscopy, scanning electron microscopy, X-ray diffraction, and mechanical property tests, the effects of different Cu contents on the microstructure, conventional mechanical properties, and low-temperature toughness at −40 °C of the steels in both as-rolled and optimally heat-treated states (solid solution at 900 °C for 1 h + aging at 540 °C for 2 h) were systematically investigated. The results demonstrate that in the as-rolled condition, with increasing Cu content, the Vickers microhardness (HV1) of the steel increases from 183.9 HV1 to 271.9 HV1, the yield strength rises from 556.55 MPa to 852.87 MPa, and the tensile strength increases from 758.53 MPa to 1162.59 MPa. Nevertheless, excessive Cu content induces austenitic grain coarsening, aggregation of Cu-rich precipitates, and stress concentration, resulting in significant deterioration of ductility and toughness. Following optimal heat treatment, the banded structure is completely eliminated, the microstructural homogeneity is substantially improved, and the ductility and toughness are remarkably enhanced compared with the as-rolled state. Meanwhile, the strength continues to increase with rising Cu content, with the 6 wt.% Cu steel achieving a yield strength of 922.51 MPa and a tensile strength of 955.17 MPa. In terms of low-temperature toughness, the 3.1 wt.% Cu steel exhibits the poorest performance (90.8 J), whereas the 6 wt.% Cu steel presents a sharply increased low-temperature impact energy of 152.6 J. This is attributed to the precipitation of particulate phases such as TiC and MnS, which effectively disperse low-temperature stress and hinder crack propagation. Overall, the CuxNi2.7Mn steel with 6 wt.% Cu possesses the highest strength as well as excellent low-temperature toughness after optimal heat treatment, providing theoretical and experimental foundations for the composition design and heat treatment process optimization of high-copper steels for marine applications.

## 1. Introduction

In recent years, copper-bearing low-carbon steel has been widely recognized in industry for its excellent comprehensive properties. It is not only extensively used in shipbuilding but also gradually applied to key engineering fields, such as bridge construction and natural gas pipelines [1,2,3]. The addition of copper to steel can significantly improve the strength, microhardness, and corrosion resistance of the material [4,5,6]. In the iron matrix, copper has a high solid solubility in γ-Fe, which decreases remarkably with decreasing temperature, providing thermodynamic conditions for precipitation strengthening during aging. In contrast, its solid solubility in α-Fe is extremely low, and excessive copper tends to segregate and precipitate at grain boundaries and dislocations [7,8,9]. The strengthening mechanisms of copper in steel mainly include solid-solution strengthening and precipitation strengthening: copper atoms in solid solution cause lattice distortion and impede dislocation motion [10,11]; finely dispersed copper-rich precipitates formed during aging can pin dislocations and grain boundaries, leading to a significant increase in strength [12,13,14]. Therefore, clarifying the microstructure evolution mechanism and mechanical property response mechanism of copper content throughout the processing and heat treatment of steel is of great theoretical significance and engineering value for optimizing the composition design of high-strength steel, fully exploiting the strengthening potential of copper, and suppressing its adverse effects.

Existing studies have shown that the strengthening effect of Cu in steel mainly comes from solid solution strengthening and the dispersed precipitation of Cu-rich phases during aging treatment. Finely distributed Cu-rich phases can effectively pin dislocations and hinder grain boundary migration, thus significantly improving the strength [15,16,17,18]. However, when the Cu content is too high, Cu-rich phases tend to coarsen and aggregate at austenite grain boundaries and dislocations, forming stress concentration sources and instead inducing brittle fracture [19,20,21,22]. Some studies have shown [23,24,25,26] that copper dissolves in austenite and reduces the stacking fault energy, inhibits dynamic recrystallization, and promotes the transformation of ferrite from continuous distribution to island/reticular morphology, and its content decreases with the increase of copper content, thus affecting microstructure evolution and crack behavior, as well as realizing the fine control of the composition and distribution of the microstructure. Moreover, the addition of Cu also affects the distribution behavior of other alloying elements in steel. For instance, the solid solution and precipitation states of Ti, Mn, Co, and other elements may change with the variation in Cu content, thereby affecting the comprehensive mechanical properties of the material [27,28,29]. However, other studies have pointed out [30,31,32,33,34] that in the relevant research on Cu-containing low-carbon steels, the Cu content is mainly concentrated at about 1~2%; there is still a lack of systematic comparison and in-depth analysis on the microstructure evolution and toughness change mechanism of steels with different Cu contents in the complete processing process, especially in the whole process from rolling, heat treatment to low-temperature service.

To fill this gap, the continuous cooling transformation (CCT) curve of CuxNi2.7Mn steel is established in this study to clarify the phase transformation kinetics and critical temperatures during heating and cooling. Combined with in situ high-temperature laser confocal microscopy, the dynamic precipitation, growth and re-dissolution behavior of Cu-rich phases are directly observed. Based on the characteristic temperatures determined by the CCT curve, the heat treatment parameters are rationally designed and verified.

Based on the above research background, this study takes CuxNi2.7Mn low-carbon steels with copper contents of 1.35 wt.%, 3.1 wt.%, and 6 wt.% as research objects, systematically investigates the effects of copper content and heat treatment temperature on the microstructure and mechanical properties, and deeply discusses the microstructure evolution characteristics and corresponding mechanisms. In situ observation and microstructure characterization methods are adopted to clarify the microstructure evolution characteristics of CuxNi2.7Mn steels with different copper contents during heating and rolling; to reveal the influence mechanism of copper content on the conventional mechanical properties (microhardness, strength, plasticity, toughness) of CuxNi2.7Mn steels in the as-rolled and heat-treated states; to explore the variation mechanism of low-temperature toughness at −40 °C of heat-treated CuxNi2.7Mn steels with different copper contents; and to determine the optimal heat treatment process that can achieve high strength and high toughness matching for high-copper CuxNi2.7Mn steels, so as to provide theoretical and experimental basis for the composition design and process optimization of high-copper marine steels.

## 2. Materials and Methods

### 2.1. Experimental Materials

The experimental process of this study was established and optimized based on the published results of the research group (Yu et al., 2025) [35]. The test steels were smelted in a vacuum melting furnace (Manufacturer, City, Country) using high-purity raw metals and selected low P and low S scrap as raw materials, with a strict charge balance to ensure accurate target composition. Before melting, deoxidation (Al + Si–Mn composite deoxidizers) and desulfurization were conducted to control inclusions and impurity elements The molten steel was cast into 200 kg ingots, and the chemical composition of the ingots was measured using a direct-reading spark spectrometer (Manufacturer, City, Country). The specific chemical composition is shown in Table 1.

The steel ingots were subsequently processed by a two-stage rolling process on a Φ450 mm two-high reversible rolling mill, with a total rolling deformation of no less than 55%. The entire rolling process was divided into two stages, with six passes set in the first stage and four passes in the second stage. The roll gap was designed in the order of original ingot → 160 mm → 135 mm → 110 mm → 85 mm → 63 mm → 45 mm → 36 mm → 29 mm → 20 mm, and the maximum reduction per pass was 25 mm. In terms of rolling process control, the first stage involves immediate continuous rolling after the billet is discharged from the furnace until the completion of the passes. The second stage begins when the steel temperature drops to 850 °C, with the finishing rolling temperature precisely controlled within the range of 800~830 °C. After rolling, laminar water cooling is employed, with a cooling water temperature of 17 °C. The total length of the cooling roller table is 6360 mm, and the roller speed is set at 0.3 m/s. Eight sets of water nozzles are arranged on both sides of the roller table, and the cooling water flow parameters are shown in Table 2.

**Figure 1:**
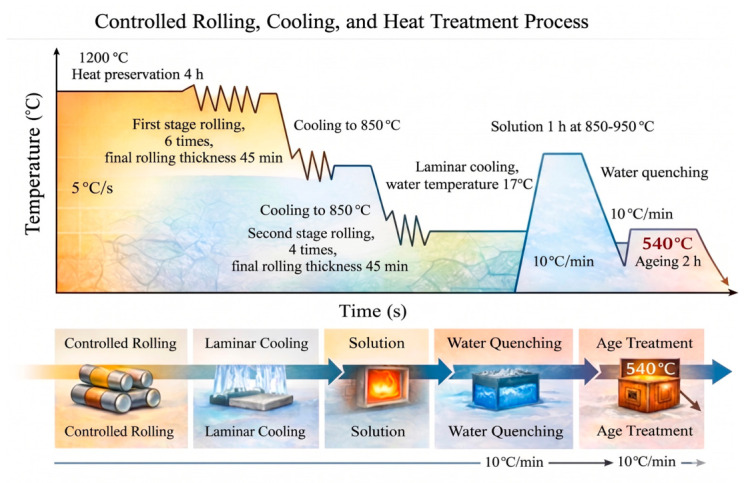
Schematic diagram of controlled rolling, controlled cooling, and heat treatment process.

After the completion of rolling and cooling, subsequent heat treatment was carried out on the test steels, and the complete controlled rolling, controlled cooling, and heat treatment process flow is shown in Figure 1. Finally, test steel samples with dimensions of 500 mm × 100 mm × 20 mm were prepared.

### 2.2. Experimental Methods

**Figure 2:**
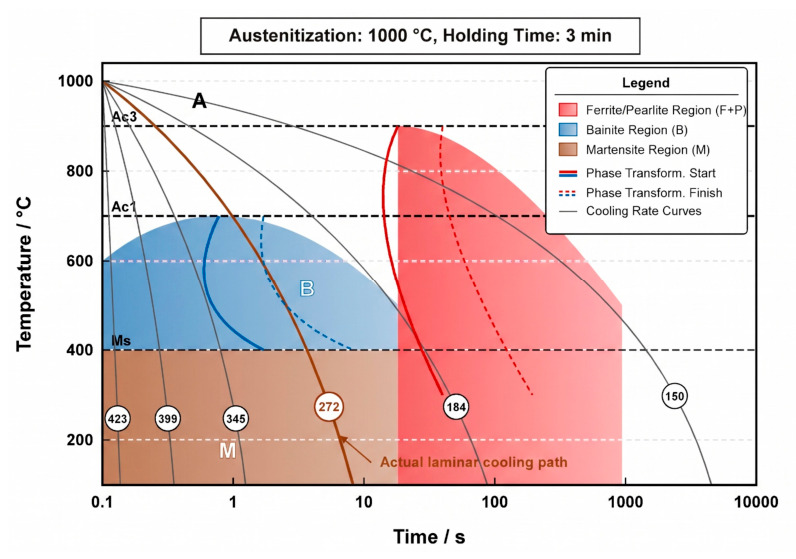
Continuous cooling transformation (CCT) curve of 6 wt.% Cu steel. A: austenite; B: bainite; M: martensite; F+P: ferrite + pearlite. Ac1, Ac3: critical phase transformation temperatures; Ms: martensite start temperature.

The continuous cooling transformation (CCT) curve of the 3# CuxNi2.7Mn steel with 6 wt.% Cu was measured by thermal dilatometry using a Formastor–FII dilatometer to determine the phase transformation kinetics, critical phase transition temperatures (Ac1, Ac3, Ms, Mf), and the characteristic temperatures corresponding to precipitation and re-dissolution of Cu-rich phases (Figure 2). In this figure, A, B, and M represent austenite, bainite, and martensite regions, respectively, and F+P denotes the ferrite + pearlite region. The key temperatures derived from the simulated CCT curve were adopted to set the heating procedure and isothermal holding points for the following in-situ high-temperature confocal observation.

**Figure 3:**
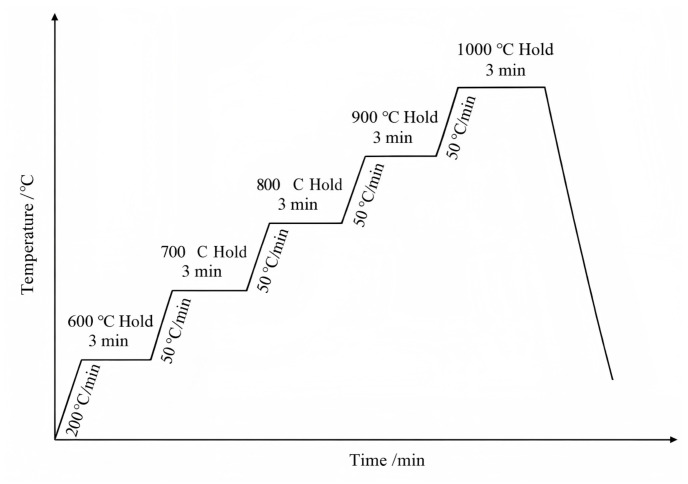
Heat treatment process flowchart.

In situ observation of the rolled samples was carried out using a high-temperature laser scanning confocal microscope (VL3000DXSVF18SP, Lasertec, Yokohama, Japan). The samples were processed into Φ7.3 mm × 3 mm, and argon gas was introduced for 3 min after vacuum extraction. Firstly, the samples were heated to 600 °C at a rate of 200 °C/min and held for 3 min; then heated to 700 °C at a rate of 50 °C/min and held for 3 min; and further heated to 800 °C, 900 °C, and 1000 °C at a rate of 50 °C/min and held for 3 min at each temperature point. The dynamic changes in the sample surface were continuously observed and recorded by the high-temperature laser scanning confocal microscope, and the corresponding high-temperature confocal heating process curve is shown in Figure 3. The test was conducted on a Vickers Q1 microhardness tester (Future-Tech Corp., Kawasaki, Japan) with a test force of 1 kgf (HV1) and a dwell time of 10 s. To achieve a more precise measurement of the overall Vickers microhardness of the sample, five points were selected on the surface of the sample (with the distance between any two points being greater than 1 mm). The final result was calculated as the average of these measurements, representing the Vickers microhardness (HV1) value of the sample.

The metallographic samples were mechanically ground step by step with SiC water sandpaper ranging from 240 # to 2000 # and then polished to a mirror surface using 3.5 μm and 1.5 μm diamond polishing agents. At room temperature, a 4% nitric acid alcohol solution (4% nitric acid by volume mixed with ethanol) was used to etch the specimens for 8–12 s to reveal the microstructure. Immediately after etching, the samples were rinsed with anhydrous ethanol and dried with hot air for subsequent observation. The microstructure after heat treatment was observed using a Zeiss Axio Vert metallographic microscope (Carl Zeiss Microscopy GmbH, Göttingen, Germany); we observed and analyzed the microstructure and spatial distribution characteristics of copper precipitates using a scanning electron microscope (SEM, ZEISS Sigma 50, Carl Zeiss Microscopy GmbH, Göttingen, Germany) equipped with an energy dispersive spectrometer (EDS). The experiment was conducted on a Vickers Q1 microhardness tester (Future-Tech Corp., Kawasaki, Japan) with a test force of 1 kgf (HV1) and a holding time of 10 s. Five measuring points were selected on the surface of each sample (with a distance between any two points greater than 1 mm), and the average value was taken as the final microhardness value. We conducted tensile testing on a universal material testing machine according to GB/T 228.1-2021 [36]. A plate-shaped tensile specimen with a thickness of 4 mm, a width of 20 mm, and a gauge length of 30 mm (specimen direction parallel to the rolling direction) was used, and the tensile rate was 3 mm/min. The yield strength, tensile strength, and elongation at break were measured. We tested three parallel samples under each process condition to ensure the repeatability of the test results. The impact test used a Charpy V-notch specimen (10 mm × 10 mm × 55 mm). The ambient temperature impact test was directly performed at room temperature (25 °C) using a pendulum impact testing machine. Low-temperature impact tests were performed at −40 °C. Specimens were immersed in a cryogenic bath and held at −40 ± 1 °C for 30 min to achieve uniform temperature throughout the cross-section. Upon reaching the target temperature, each specimen was rapidly transferred to the impact tester, and the test was completed within 3 s to minimize temperature drift. Three replicate tests were conducted for each condition, and the average impact energy was reported. Fracture surfaces were subsequently examined by scanning electron microscopy (SEM) to characterize the fracture morphology and identify the operative fracture mechanisms.

## 3. Results and Discussion

### 3.1. Microstructure

#### 3.1.1. In Situ Observation by High-Temperature Confocal Microscopy

**Figure 4:**
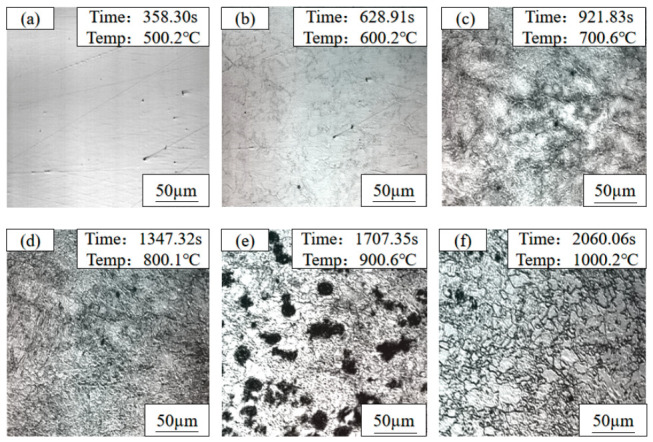
In situ observation micrographs of 1# (1.35 wt.% Cu) copper-bearing steel during heat treatment: (**a**) 500 °C; (**b**) 600 °C; (**c**) 700 °C; (**d**) 800 °C; (**e**) 900 °C; (**f**) 1000 °C.

**Figure 5:**
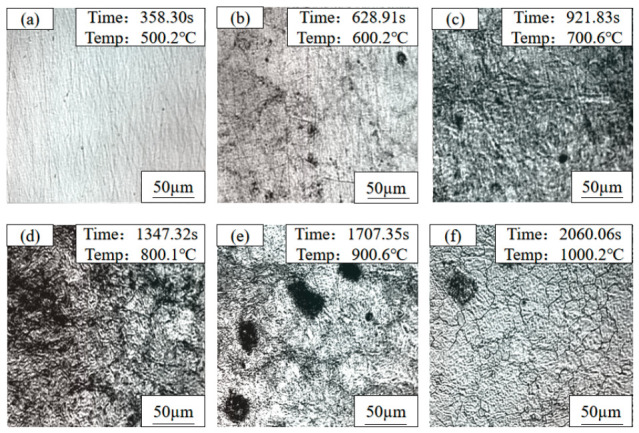
In situ observation micrographs of 2# (3.1 wt.% Cu) copper-bearing steel during heat treatment: (**a**) 500 °C; (**b**) 600 °C; (**c**) 700 °C; (**d**) 800 °C; (**e**) 900 °C; (**f**) 1000 °C.

**Figure 6:**
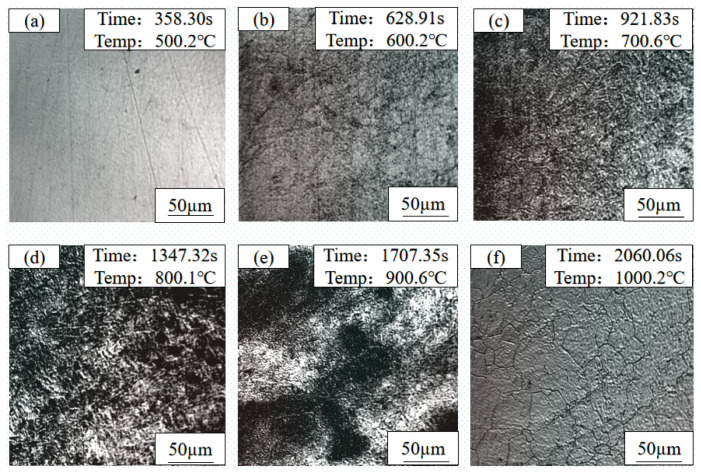
In situ observation micrographs of 3# (6 wt.% Cu) copper-bearing steel during heat treatment: (**a**) 500 °C; (**b**) 600 °C; (**c**) 700 °C; (**d**) 800 °C; (**e**) 900 °C; (**f**) 1000 °C.

To quantitatively reveal the kinetic evolution of microstructure and Cu-rich precipitates during continuous heating, and to verify the phase transformation sequence determined by the CCT curve, in situ observations were conducted on three as-rolled test steels (1#, 2#, and 3#) using a high-temperature confocal laser scanning microscope. The temperature points for in situ observation were strictly selected according to the characteristic temperatures determined by the CCT curve. The microstructures and morphologies of the three steels at different temperatures are shown in Figure 4, Figure 5 and Figure 6, respectively.

For the 1# test steel (1.35 wt.% Cu), at 500 °C, the microstructure showed no significant changes, with unclear grain boundaries and a relatively smooth sample surface. When heated to 600 °C, blurred polygonal grain boundary contours began to appear on the sample surface, accompanied by a small amount of fine black phases precipitating at the grain boundaries. At 700 °C, the grain boundary contours became clearer, and the amount of black precipitates increased slightly but remained sparsely distributed. At 800 °C, the grain boundaries were fully visible, and the number of black precipitates reached its peak, which were small in size and uniformly distributed along the grain boundaries. During holding at 900 °C, the black precipitates began to enrich and coalesce, forming small clusters in some regions. After holding at 1000 °C, all black precipitates were completely dissolved back into the austenite matrix, with clear austenite grain boundaries and fine, uniform grain size.

For the 2# test steel (3.1 wt.% Cu), there was no obvious microstructure change at 500 °C. After holding at 600 °C, the grain boundaries began to appear, and the number of black precipitates was slightly higher than that of the 1# steel. At 700 °C, the grain boundaries were clearer, the number of precipitates increased significantly, and the distribution was denser. At 800 °C, the number of precipitates reached a peak; the grain boundaries were almost covered with fine black particles, and slight enrichment occurred in some areas. During holding at 900 °C, the enrichment and merging of precipitates were more significant, forming large-sized clusters at the grain boundary intersections. After holding at 1000 °C, the precipitates were completely dissolved back, and the austenite grain size was significantly larger than that of the 1# steel, with straight and clear grain boundaries.

For the 3# test steel (6 wt.% Cu), there was also no obvious microstructure change at 500 °C. After holding at 600 °C, the grain boundaries appeared, and the number of black precipitates was significantly higher than that of the other two steels. At 700 °C, the number of precipitates increased sharply. They were distributed not only along the grain boundaries, but fine precipitation points also appeared in some intragranular regions. At 800 °C, the number of precipitates reached the maximum. The grain boundaries were covered with a dense layer of black precipitates and formed obvious precipitation bands, and a large number of finely dispersed precipitates also existed in the grains. During holding at 900 °C, the precipitates significantly enriched and coarsened, forming large-sized clusters, and the grain boundaries became thick. After holding at 1000 °C, the precipitates were completely dissolved back, and the austenite grain size was significantly larger than that of the 1# and 2# steels, showing an obvious grain coarsening morphology.

The microstructure evolution of the three test steels during heating were roughly consistent. At 500 °C, the microstructure differences among the three steels were small; with the temperature rising to 600 °C and 700 °C, the grain boundary contours gradually appeared, and black substances precipitated on the sample surface, with the number increasing gradually. At 800 °C, the number of black precipitates increased significantly, while during holding at 900 °C, the black precipitates began to enrich. In comparison, the higher the Cu content, the greater the number of black precipitates; when the temperature rose to 1000 °C and held, all black precipitates in the test steels were completely dissolved back into the matrix, and the austenite grain boundaries were clearly visible.

By correlating the in situ results with the CCT curve of CuxNi2.7Mn steel, the precipitation peak temperature of Cu-rich phases (≈800 °C) and critical re-dissolution temperature (≈1000 °C) were accurately determined, together with the austenite grain growth kinetics. The stepwise heating under Ar protection reproduced the core metallurgical reactions (precipitation, re-dissolution, grain growth) occurring in the heating stage of the solution treatment under air atmosphere. Despite slight differences in heating rate and atmosphere, the key phase transition temperatures and Cu-rich phase evolution mechanisms were consistent. Therefore, the confocal in situ observations provide direct kinetic evidence for understanding microstructural evolution during solution treatment, and support the selection of the solution temperature in the heat treatment process.

#### 3.1.2. Effect of Different Copper Contents on Microstructure and Properties of As-Rolled Steel

**Figure 7:**
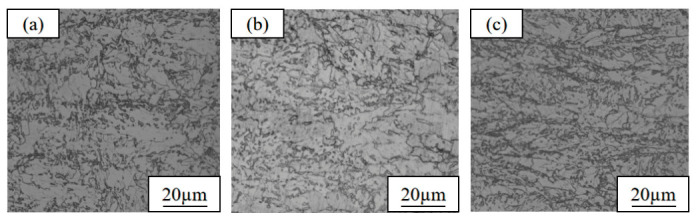
Metallographic micrographs of copper-bearing steels with different copper contents in as-rolled state: (**a**) 1# (1.35 wt.% Cu); (**b**) 2# (3.1 wt.% Cu); (**c**) 3# (6 wt.% Cu).

The metallographic microstructures of the three as-rolled test steels with different Cu contents are shown in Figure 7. It can be seen that the microstructures of the three test steels are mainly composed of ferrite and granular bainite, and banded structures are present due to the non-uniform stress during rolling. With increasing Cu content, the proportion of granular bainite in the microstructure exhibits a gradual increasing trend. Among them, steel 1# (1.35 wt.% Cu) contains a small amount of granular bainite, with ferrite as the dominant phase. The content of granular bainite in steel 3# (6 wt.% Cu) is significantly increased, making it one of the dominant microstructures. Such differences in the microstructure composition directly lead to the distinct differences in the mechanical properties of the steels.

The test results of the mechanical properties of the test steels in rolled states are shown in Table 3. The microhardness, strength, ductility and toughness indices show significant regularity with the change in Cu content, and the change is mainly affected by the synergistic effect of the solid solution strengthening and precipitation strengthening of Cu and microstructure evolution. In terms of strength and microhardness indices, with the increase in Cu content, the Vickers microhardness (HV1), yield strength and tensile strength of the test steels all show a continuous increasing trend: the microhardness of 1# steel is 183.9 HV1, the yield strength is 556.55 MPa, and the tensile strength is 758.53 MPa, which are the lowest values among the three; the strength indices of 2# steel are significantly improved, with the microhardness, yield strength, and tensile strength increasing to 243.1 HV1, 788.14 MPa, and 1058.01 MPa, respectively; and the strength and microhardness of 3# steel reach their peaks, with the microhardness of 271.9 HV1, the yield strength of 852.87 MPa, and the tensile strength of 1162.59 MPa.

The continuous strength enhancement of the test steels in the as-rolled state is primarily attributed to three mechanisms: First, the solid solution strengthening effect of Cu:when Cu atoms dissolve into the iron matrix, they induce lattice distortion that significantly impedes dislocation motion [37,38]. Notably, the degree of lattice distortion increases with increasing Cu content, leading to a more pronounced solid solution strengthening effect. Second, the precipitation strengthening during the rolling process—the thermal deformation and subsequent cooling in rolling promote the formation of supersaturated solid solutions in the steel. Cu atoms tend to precipitate as fine Cu-rich phases at defects such as dislocations and grain boundaries. These precipitates exert a strong “pinning” effect on dislocation slip, thereby further improving the material strength. With the increase in Cu content, the supersaturation degree of the solid solution is enhanced, resulting in a higher volume fraction of precipitates and a more prominent contribution of precipitation strengthening. Third, the influence of microstructure composition—steel 3# exhibits the highest proportion of granular bainite among the three steels. Given that granular bainite inherently possesses high microhardness and strength, it becomes a key microstructural factor responsible for the significantly higher strength of steel 3# compared to steels 1# and 2#.

In contrast to the increasing trend of strength and microhardness, the ductility and toughness of the tested steel exhibit a continuous deterioration with the increase in Cu content, and the degree of deterioration gradually intensifies with higher Cu addition. Steel 1# shows the best ductilityand toughness, with an elongation of 27.7% and a room-temperature impact (RTI) energy of 154.9 J. Steel 2# presents a significant decrease in ductility and toughness, where the elongation drops to 21.8% and the RTI energy is only 53.3 J. Steel 3# exhibits obvious brittle characteristics, with the elongation falling to 17.4% and the RTI energy merely 29.2 J, representing the lowest ductility and toughness among the three steels.

**Figure 8:**
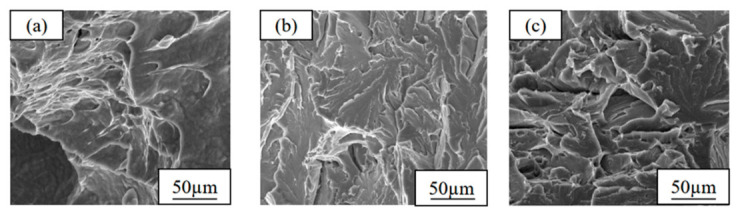
Fracture morphologies of copper-bearing steels with different copper contents in as-rolled states: (**a**) 1# (1.35 wt.% Cu); (**b**) 2# (3.1 wt.% Cu); (**c**) 3# (6 wt.% Cu).

The analysis results of the impact fracture morphology of the as-rolled test steels further verify the above mechanical property variation law (Figure 8): a large number of dimples with varying sizes are distributed on the fracture surface of steel 1#, with large dimple depths, showing typical ductile fracture characteristics; the number of dimples on the fracture surface of steel 2# is significantly reduced, and numerous river-like cleavage planes appear, with the fracture mode between ductile and brittle fracture; the fracture morphology of steel 3# tends to be flat, the dimple structure almost completely disappears, and the fracture is dominated by cleavage fracture, showing obvious brittle fracture characteristics.

The fundamental reason for this change in fracture morphology lies in the thermodynamic instability of the supersaturated solid solution in the as-rolled state and the inhomogeneous precipitation of Cu-rich phases at grain boundaries and dislocations during cooling [39,40]. It can be seen that although the strengthening effect of Cu on the as-rolled steel is very significant, excessively high Cu content will lead to issues such as microstructural coarsening, precipitation aggregation, and aggravated stress concentration, thereby seriously deteriorating the ductility and toughness of the material. Therefore, regulating the precipitation behavior of Cu and optimizing microstructural uniformity through subsequent heat treatment are the keys to achieving the synergy of high strength and toughness in high-Cu steels.

### 3.2. Microstructure and Mechanical Properties in Heat-Treated State

**Figure 9:**
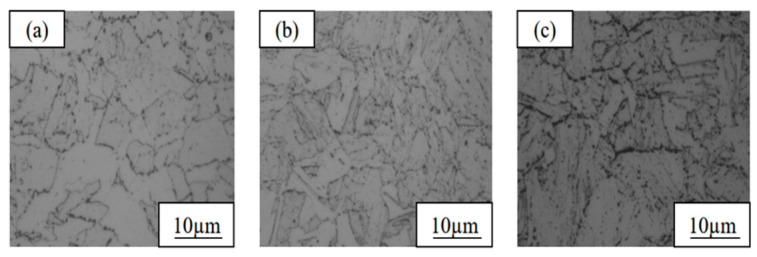
Observation micrographs under the optimal process (900 °C-ST × 1 h + 540 °C-AG × 2 h): (**a**) 1# (1.35 wt.% Cu); (**b**) 2# (3.1 wt.% Cu); (**c**) 3# (6 wt.% Cu).

The microstructural observations of the three experimental steels after the optimal heat treatment process are shown in Figure 9. The optimal heat treatment process consists of solution treatment at 900 °C for 1 h, followed by aging at 540 °C for 2 h (900 °C-ST × 1 h + 540 °C-AG × 2 h) [35]. Compared with the as-rolled state, the banded structures of the test steels after heat treatment are completely eliminated; the microstructure uniformity is significantly improved, and the microstructure mainly comprises ferrite, lath bainite, and a small amount of retained austenite. However, obvious differences in the phase constitution exist among the test steels with different Cu contents: the microstructure of steel 1# (1.35 wt.% Cu) is dominated by ferrite, with lath bainite uniformly and dispersively distributed; the content of lath bainite in steel 2# (3.1 wt.% Cu) relatively increases, ferrite grains grow slightly, and a small number of fine precipitated phases are observed at the grain boundaries; the ferrite content in steel 3# (6 wt.% Cu) is significantly higher than that of the other two steels, the lath bainite content relatively decreases, ferrite grain aggregation occurs in some regions, and a small amount of undissolved precipitated phases remains at the grain boundaries.

#### 3.2.1. Conventional Mechanical Properties

**Figure 10:**
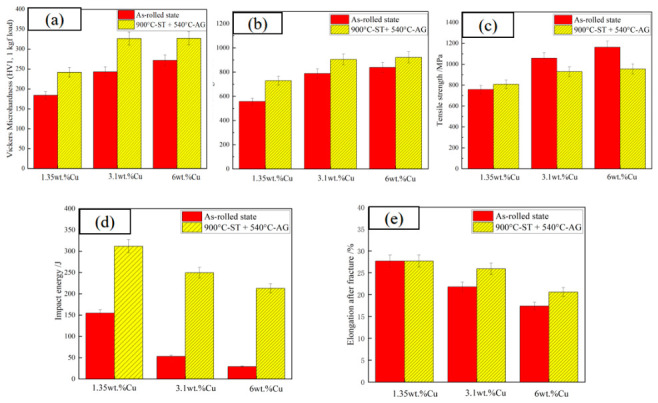
Comparison of mechanical properties between as-rolled state and heat-treated state: (**a**) Vickers microhardness (HV1, 1 kgf load); (**b**) yield strength; (**c**) tensile strength; (**d**) RTI energy; (**e**) elongation.

The comparison results of microhardness, yield strength, tensile strength, room-temperature impact energy, and elongation of the experimental steels with different Cu contents in both the as-rolled state and the heat-treated state (900 °C-ST × 1 h + 540 °C-AG × 2 h) are shown in Figure 10. All mechanical property indices exhibit significant regularity with variations in Cu content. Moreover, the comprehensive mechanical properties of the experimental steels after heat treatment are significantly optimized compared to those in the as-rolled state, and the strength–toughness matching is effectively improved.

Vickers microhardness (HV1) (Figure 10a): The microhardness in the heat-treated state increases continuously with the increase in Cu content and is higher than that in the rolled state. The microhardness of steel 1# increases from 183.9 HV1 to about 200 HV1, steel 2# from 243.1 HV1 to about 250 HV, and steel 3# from 271.9 HV1 to about 300 HV. This is the result of the synergistic effect of solid solution strengthening of Cu and dispersion precipitation strengthening of Cu-rich phases, and the higher the Cu content, the more significant the strengthening effect.

Yield strength (Figure 10b): The yield strength in the heat-treated state shows a continuous increasing trend and is higher than that in the rolled state. The yield strength of steel 1# increases from 556.55 MPa to about 700 MPa, steel 2# from 788.14 MPa to about 800 MPa, and steel 3# from 852.87 MPa to 922.51 MPa, indicating that heat treatment does not weaken the strengthening effect of Cu, but also improves the plastic deformation resistance of the material by eliminating internal stress and optimizing the microstructure.

Tensile strength (Figure 10c): The tensile strength in the heat-treated state shows an increasing trend with the increase in Cu content, but the high-Cu samples slightly decrease while the low-Cu samples increase. The tensile strength of steel 1# increases from 758.53 MPa to about 800 MPa, steel 2# from 1058.01 MPa to about 950 MPa, and steel 3# from 1162.59 MPa to 955.17 MPa, which is still the highest value in the heat-treated state.

RTI energy (Figure 10d): The room-temperature impact energy in the heat-treated state decreases gradually with the increase in Cu content, but is significantly higher than that in the rolled state. The impact energy of steel 1# increases from 154.9 J to 312.3 J, steel 2# from 53.3 J to about 250 J, and steel 3# from 29.2 J to 213.3 J, realizing a qualitative improvement in toughness.

Elongation (Figure 10e): The change law is consistent with that of room-temperature impact energy, and the elongation in the heat-treated state is higher than that in the rolled state. The elongation of steel 1# slightly increases from 27.7% to 28.0%, steel 2# from 21.8% to about 25%, and steel 3# from 17.4% to 20.8%, with the plastic deterioration problem significantly alleviated.

The strength improvement under the heat-treated state originates from the synergistic effect of solid solution strengthening and precipitation strengthening of Cu. The solid solution treatment enables Cu-rich phases to redissolve into the matrix lattice, inducing lattice distortion and hindering dislocation motion. Meanwhile, aging treatment promotes the precipitation of fine Cu-rich particles, which exert a pinning effect on dislocation slip. The slight decrease in the tensile strength of steels 2# and 3# is because the higher the Cu content, the faster the precipitation of Cu-rich phases at the grain boundaries, which reduces the grain boundary bonding strength and easily induces intergranular microcracks during stretching. In contrast, the yield strength is less affected by the grain boundaries, thus maintaining an increasing trend.

The general improvement of ductility and toughness after heat treatment is due to the elimination of rolled banded structure, the release of internal stress by the process, and the transformation of Cu-rich phases from inhomogeneous aggregation to uniform dispersion precipitation, alleviating the stress concentration; meanwhile, the ductility and toughness still decrease with the increase in Cu content because the high-Cu content leads to an increase in the precipitation amount and a slight increase in the size of Cu-rich phases form local stress concentration, though this effect is much weaker than that in the rolled state, and the ductility and toughness of the samples all meet the engineering requirements.

#### 3.2.2. Mechanical Property Analysis

**Figure 11:**
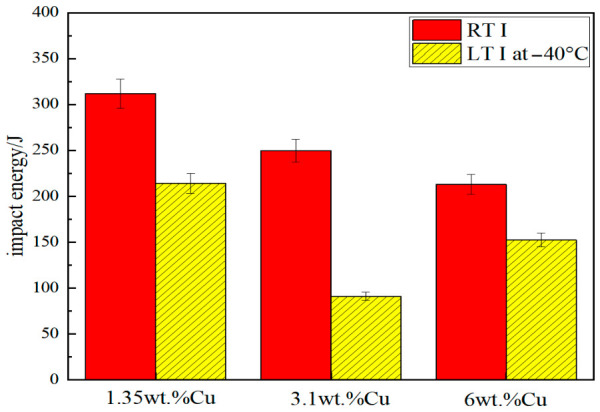
RTI and LTI toughness test results of heat-treated test steels with different Cu contents.

Considering the application requirements of Cu-bearing steels in marine engineering and other fields, LTI tests at −40 °C were conducted on the heat-treated experimental steels, and the test results are shown in Figure 11. Compared with the RTI energy, the impact energy of the three experimental steels decreases to varying degrees at low temperatures. This is attributed to the weakened thermal motion of atoms at low temperatures, which restricts dislocation slip and thus leads to an overall reduction in material toughness [41,42].

The LTI energy of the test steel 1# is 213.9 J, maintaining a high level, which is the sample with the best low-temperature toughness among the three; the LTI energy of the test steel 2# drops to 90.8 J, the minimum value, with the toughness significantly decreased; while the LTI energy of the test steel 3# rebounds sharply to 152.6 J, much higher than that of the test steel 2#. Except for the test steel 2#, the LTI energy of the other test steels remains at a high level of more than 150 J.

**Figure 12:**
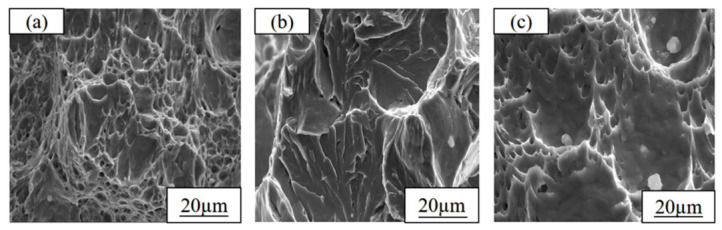
LTI fracture morphologies of Cu-bearing steels under the optimal heat treatment condition: (**a**) 1# (1.35 wt.% Cu); (**b**) 2# (3.1 wt.% Cu); (**c**) 3# (6 wt.% Cu).

To reveal the change mechanism of low-temperature toughness, scanning electron microscope observation was conducted on the LTI fracture morphologies of the three test steels at −40 °C, and it was found that the fracture characteristics were highly correlated with the low-temperature toughness (Figure 12): a large number of dimples with different sizes are distributed on the fracture of the test steel 1#, with only a small amount of river-like tear ridges existing near the dimples, and the fracture mode is dominated by ductile fracture with larger dimple depth and better fracture resistance; there are almost no dimple structures on the fracture of the test steel 2#, which is completely dominated by tear ridges, showing typical brittle fracture characteristics, which is the direct reason for its worst low-temperature toughness; the fracture of the test steel 3# is again dominated by dimples, with dimples taking large dimples as the core and a large number of small dimples distributed around, and polygonal granular precipitates exist in the large dimples, and the fracture mode recovers to ductile fracture, resulting in a significant improvement in its low-temperature toughness.

**Figure 13:**
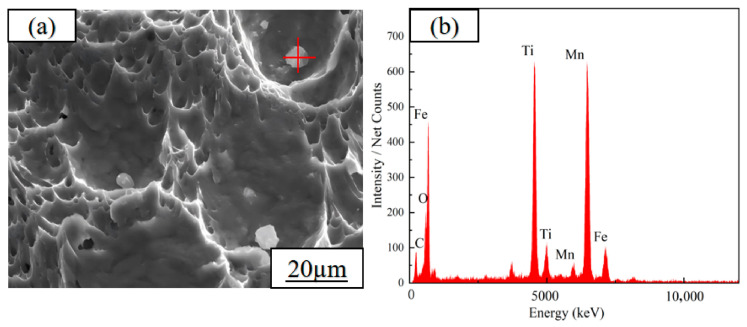
(**a**) SEM morphology of precipitates on −40 °C impact fracture of 3# steel (red “+”: EDS point); (**b**) corresponding EDS spectrum.

Energy dispersive spectroscopy (EDS) analysis was carried out on the polygonal granular precipitates in the fracture of the 3# test steel, and the results show that the precipitates are mainly composed of Ti and Mn, which are compounds such as TiC and MnS (Figure 13). Figure 13a shows the SEM morphology of the precipitate, where the red “+” marks the EDS point scanning position. Figure 13b presents the corresponding EDS spectrum. The formation of such precipitated phases is closely related to the Cu content: when the Cu content is low, the interaction between solute atoms such as Ti and Mn and the iron matrix is strong, existing in the matrix in the form of solid solution and unable to form precipitated phases; while with the increase of Cu content, this interaction is weakened, Ti and Mn cannot be completely dissolved, and form granular precipitates such as TiC during heat treatment and low-temperature environment. When TiC particles are dispersed in the metal matrix, they can provide strong local constraints, effectively disperse the internal stress at low temperatures, hinder the initiation and propagation of cracks, thus reducing the risk of brittle fracture, which is the key reason for the rebound of low-temperature toughness of the 3# steel. At the same time, the overall free energy of the system decreases at low temperatures, promoting the easier combination of Ti and C to form TiC, which is also an important factor that the precipitated phase is not found in the room-temperature fracture of the 3# steel but only exists in the low-temperature fracture.

### 3.3. Evolution Law of Microstructure and Properties of CuxNi2.7Mn Steel and Regulation Mechanism of Cu

**Figure 14:**
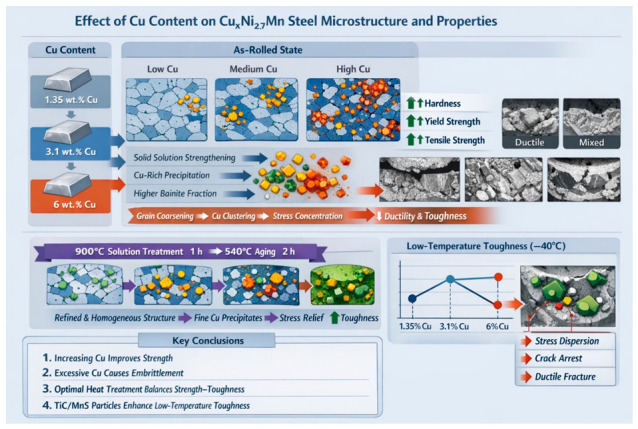
Microstructure property evolution mechanism diagram of CuxNi2.7Mn steel.

The microstructure and mechanical properties of CuxNi2.7Mn steels are highly dependent on the Cu content. The underlying mechanisms differ significantly between the as-rolled and heat-treated conditions, in which solid solution strengthening, precipitation strengthening, and grain refinement induced by Cu play dominant roles. Heat treatment can achieve superior strength–toughness synergy via microstructural optimization. In addition, the precipitation of special secondary phases at high Cu content contributes to a unique low-temperature toughening mechanism. The overall evolution characteristics is illustrated in Figure 14.

In the as-rolled state, the strength increases continuously with rising Cu content due to solid solution strengthening, precipitation strengthening, and an increased fraction of granular bainite. However, high Cu addition leads to austenite grain coarsening and inhomogeneous segregation of Cu-rich phases at grain boundaries, which, together with the intensified heterogeneity caused by banded structures, induces severe stress concentration and thus drastically deteriorates ductility and toughness.

After heat treatment consisting of 900 °C-ST × 1 h + 540 °C-AG × 2 h, internal stress and banded structures are eliminated, and Cu-rich phases are redissolved during solution treatment. Subsequent aging promotes the uniform and dispersed precipitation of Cu-rich phases. Consequently, the strengthening effect of Cu is retained, and the strength still increases with Cu content. Meanwhile, stress concentration is effectively alleviated, leading to remarkably improved ductility and toughness compared with the as-rolled state, thereby achieving optimized strength–toughness synergy. The slight decrease in ultimate tensile strength at high Cu levels is attributed to the rapid precipitation of Cu-rich phases at grain boundaries, which slightly reduces grain boundary cohesion.

The low-temperature toughness at −40 °C of heat-treated CuxNi2.7Mn steels varied nonlinearly with Cu content. The 3.1 wt.% Cu steel showed the lowest low-temperature impact energy of 90.8 J. In contrast, the 6 wt.% Cu steel exhibited a rebound in low-temperature impact energy to 152.6 J. The elevated Cu content weakens the affinity between Ti and Mn solute atoms and the Fe matrix, which reduces the solid solubility of Ti and Mn in the austenite matrix during solution treatment and promotes the nucleation and growth of TiC and MnS particles during subsequent aging and cooling [43,44]. These fine dispersed particles act as effective crack arrestors as, on one hand, they can disperse local stress concentration at low temperatures and prevent the initiation of intergranular and transgranular cracks; on the other hand, they can pin crack tips and force cracks to deflect or branch during propagation, thereby increasing crack propagation energy and significantly improving low-temperature toughness [45,46]. This reveals the unique low-temperature toughening mechanism of high-copper steels.

Overall, Cu acts as the core contributor to the enhanced strength of CuxNi2.7Mn steels, while its embrittling effect can be eliminated by appropriate heat treatment. The precipitation of TiC and MnS induced by high Cu content provides a critical strategy for tailoring the low-temperature properties of high copper offshore engineering steels.

## 4. Conclusions

In this study, through the regulation of rolling and heat treatment processes of CuxNi2.7Mn steels with different Cu contents (1.35 wt.%, 3.1 wt.% and 6 wt.%), combined with a variety of microstructural characterization and mechanical property tests, the influence mechanism of Cu content on the microstructure evolution and mechanical properties of this steel grade was deeply explored, and the main conclusions are as follows:

(1) The strength enhancement of as-rolled CuxNi2.7Mn steels originates from the synergistic effects of Cu solid solution strengthening, precipitation strengthening, and an increased proportion of granular bainite. As the Cu content increased from 1.35 wt.% to 6 wt.%, the yield strength rose from 556.55 MPa to 852.87 MPa, and the tensile strength increased from 758.53 MPa to 1162.59 MPa. Nevertheless, excessive Cu content led to austenite grain coarsening, inhomogeneous aggregation of Cu-rich precipitates, and aggravated banded structure, resulting in significant deterioration of ductility and toughness. The 6 wt.% Cu steel exhibited an elongation of only 17.4% and a room-temperature impact energy of merely 29.2 J, demonstrating typical brittle fracture characteristics.
(2) The optimal heat treatment process for CuxNi2.7Mn steels was determined as solution treatment at 900 °C for 1 h followed by aging at 540 °C for 2 h. This process completely eliminated the as-rolled banded structure, relieved internal stress, refined grains, and promoted uniform and dispersed precipitation of Cu-rich phases. While retaining the Cu strengthening effect and maintaining a continuous increase in strength with rising Cu content, the ductility and toughness were remarkably improved compared with the as-rolled state, realizing the synergistic optimization of strength and toughness.
(3) The low-temperature toughness at −40 °C of heat-treated CuxNi2.7Mn steels varied nonlinearly with Cu content. The 3.1 wt.% Cu steel showed the lowest low-temperature impact energy of 90.8 J. In contrast, the 6 wt.% Cu steel exhibited a rebound in low-temperature impact energy to 152.6 J. This was attributed to the fact that high Cu content weakened the interaction of Ti and Mn with the matrix, facilitating the precipitation of TiC and MnS particles, which effectively dispersed low-temperature stress and hindered crack propagation, revealing the unique low-temperature toughening mechanism of high-copper steels.
(4) The CCT curve combined with in situ high-temperature confocal observation accurately reveals the kinetic characteristics of Cu-rich phase precipitation and re-dissolution during heating, which provides a solid kinetic basis for the determination of solution temperature and the optimization of the entire heat treatment system for high-copper marine engineering steels.
(5) After the optimal heat treatment, the CuxNi2.7Mn steel with 6 wt.% Cu possessed the best comprehensive performance, featuring the highest strength, favorable ductility, and excellent low-temperature toughness. It is verified that reasonable heat treatment can circumvent the embrittlement effect induced by high Cu content and fully exploit its strengthening potential, providing a direct basis for composition design and process regulation of high-copper steels for marine engineering applications.

## Figures and Tables

**Table 1:** Chemical compositions of the three steels (wt.%).

Steel	Cu	C	Ni	Mn	Cr	Si	Ti	S	Fe
1#	1.35	0.027	2.71	0.97	0.48	0.19	0.012	0.0019	Bal.
2#	3.1	0.021	2.72	0.95	0.48	0.18	0.011	0.0019	Bal.
3#	6	0.036	2.7	0.97	0.48	0.2	0.0089	0.0012	Bal.

**Table 2:** Cooling water settings (m^3^/h).

Pass	1	2	3	4	5	6	7	8
Above	35	35	35	35	35	35	35	35
Below	40	40	35	35	35	40	40	40

**Table 3:** Mechanical property test results of as-rolled steels with different copper contents (*n* = 3, mean ± SD).

Sample	Vickers Microhardness/HV1	Yield Strength/MPa	Tensile Strength/MPa	RTI Energy/J	Elongation/%
1#	183.9 ± 4.2	556.55 ± 10.3	758.53 ± 12.6	154.9 ± 5.8	27.7 ± 1.1
2#	243.1 ± 5.6	788.14 ± 13.5	1058.01 ± 18.2	53.3 ± 2.4	21.8 ± 0.9
3#	271.9 ± 6.1	852.87 ± 14.8	1162.59 ± 20.5	29.2 ± 1.3	17.4 ± 0.7

## Data Availability

The original contributions presented in this study are included in the article. Further inquiries can be directed to the corresponding author.
